# Efficacy of Adjuvant Interferon‐β Versus Observation for Resected Nail Apparatus Melanoma: A Multicenter Retrospective Study

**DOI:** 10.1111/1346-8138.70375

**Published:** 2026-07-06

**Authors:** Takaya Komori, Eiji Nakano, Yukiko Kiniwa, Shoichiro Mori, Sotaro Yamamoto, Hiroshi Kato, Shusuke Yoshikawa, Koji Yoshino, Megumi Aoki, Tatsuya Takenouchi, Takuya Maeda, Keijun Yoshino, Shuichi Ohe, Kenta Nakama, Hideyuki Ishikawa, Yoshiyuki Nakamura, Toshihiro Takai, Takamichi Ito, Hiroshi Kitagawa, Hiraku Kokubu, Naohito Hatta, Takeru Funakoshi, Toshihiko Hoashi, Takayuki Suyama, Susumu Fujiwara, Mamiko Masuzawa, Hiroshi Uchi, Takuya Miyagawa, Soichiro Kado, Yuki Yamamoto, Jun Asai, Junji Kato, Taku Maeda, Natsuki Baba, Yukihiko Kato, Kohei Oashi, Takeo Maekawa, Shunichi Jinnai, Aya Nishizawa, Ko Kagoyama, Atsushi Otsuka, Katsuhiko Nishihara, Dai Ogata, Kenjiro Namikawa, Yasuhiro Nakamura, Shigeto Matsushita

**Affiliations:** ^1^ Department of Skin Oncology/Dermatology Saitama Medical University International Medical Center Saitama Japan; ^2^ Department of Dermatology Kindai University Faculty of Medicine Osaka Japan; ^3^ Department of Dermatologic Oncology National Cancer Center Hospital Tokyo Japan; ^4^ Department of Dermatology Shinshu University School of Medicine Matsumoto Japan; ^5^ Department of Dermatology Nagoya University Graduate School of Medicine Nagoya Japan; ^6^ Department of Dermatology and Plastic Surgery, Faculty of Life Sciences Kumamoto University Kumamoto Japan; ^7^ Department of Geriatric and Environmental Dermatology Nagoya City University Graduate School of Medical Sciences Nagoya Japan; ^8^ Department of Dermatology Shizuoka Cancer Center Shizuoka Japan; ^9^ Department of Dermato‐Oncology/Dermatology Cancer Institute Hospital of Japanese Foundation for Cancer Research Tokyo Japan; ^10^ Department of Dermato‐Oncology NHO Kagoshima Medical Center Kagoshima Japan; ^11^ Division of Dermatology Niigata Cancer Center Hospital Niigata Japan; ^12^ Department of Dermatology, Faculty of Medicine, Graduate School of Medicine Hokkaido University Sapporo Japan; ^13^ Department of Dermatology Chiba University Graduate School of Medicine Chiba Japan; ^14^ Department of Dermatologic Oncology Osaka International Cancer Institute Osaka Japan; ^15^ Department of Dermatology Kurume University School of Medicine Kurume Japan; ^16^ Department of Environmental Immuno‐Dermatology Yokohama City University Graduate School of Medicine Kanagawa Japan; ^17^ Department of Dermatology Institute of Medicine, University of Tsukuba Tsukuba Japan; ^18^ Department of Dermatology Hyogo Cancer Center Akashi Japan; ^19^ Department of Dermatology, Graduate School of Medical Sciences Kyushu University Fukuoka Japan; ^20^ Department of Dermatology, Graduate School of Medicine Mie University Tsu Japan; ^21^ Department of Dermatology Shiga University of Medical Science Otsu Japan; ^22^ Department of Dermatology Toyama Prefectural Central Hospital Toyama Japan; ^23^ Department of Dermatology Keio University School of Medicine Tokyo Japan; ^24^ Department of Dermatology Nippon Medical School Tokyo Japan; ^25^ Department of Dermatology Dokkyo Medical University Saitama Medical Center Saitama Japan; ^26^ Division of Dermatology, Department of Internal Related Kobe University Graduate School of Medicine Kobe Japan; ^27^ Department of Dermatology Kitasato University School of Medicine Kanagawa Japan; ^28^ Department of Dermato‐Oncology NHO Kyushu Cancer Center Fukuoka Japan; ^29^ Department of Dermatology The University of Tokyo Graduate School of Medicine Tokyo Japan; ^30^ Department of Dermatology Jichi Medical University Tochigi Japan; ^31^ Department of Dermatology Wakayama Medical University Wakayama Japan; ^32^ Department of Dermatology Kyoto Prefectural University of Medicine Graduate School of Medical Science Kyoto Japan; ^33^ Department of Dermatology Sapporo Medical University School of Medicine Sapporo Japan; ^34^ Department of Plastic and Reconstructive Surgery, Faculty of Medicine and Graduate School of Medicine Hokkaido University Sapporo Japan; ^35^ Department of Dermatology, Division of Medicine, Faculty of Medical Sciences University of Fukui Fukui Japan; ^36^ Department of Dermatology, Hachioji Medical Center Tokyo Medical University Hachioji Japan; ^37^ Department of Dermatology Saitama Cancer Center Saitama Japan; ^38^ Department of Dermatology, Saitama Medical Center Jichi Medical University Saitama Japan; ^39^ Dermatologic Oncology National Cancer Center Hospital East Kashiwa Japan; ^40^ Department of Dermatology, Tokyo Metropolitan Cancer and Infectious Disease Center Komagome Hospital Tokyo Japan; ^41^ Department of Dermatology Academic Assembly University of Toyama Toyama Japan; ^42^ Department of Dermatology Ehime University Graduate School of Medicine Toon Japan

**Keywords:** adjuvant therapy, interferon, interferon‐β, nail apparatus melanoma

## Abstract

Nail apparatus melanoma (NAM) is a biologically and clinically distinct subtype of melanoma; however, the efficacy of adjuvant interferon (IFN) therapy in this population remains unclear. We conducted a multicenter retrospective study to compare adjuvant IFN‐β (adj‐IFN) with observation (OBS) in patients with resected stage IIB, IIC, or III NAM. We analyzed 282 Japanese patients treated at 42 institutions between 2014 and 2024. Recurrence–free survival (RFS) and distant metastasis–free survival (DMFS) were co‐primary outcomes, and overall survival (OS) was a secondary outcome. Survival outcomes were assessed using Kaplan–Meier analyses and Cox multivariable proportional hazards models. To minimize the differences of baseline characteristics between the two groups, propensity score matching (PSM) was applied. In multivariable Cox analyses, adj‐IFN showed improved RFS (HR 0.67, 95% CI 0.45–0.998, *p* = 0.049), while no statistically significant benefits were observed for DMFS (HR 0.66, 95% CI 0.42–1.01, *p* = 0.06) or OS (HR 0.85, 95% CI 0.54–1.35, *p* = 0.50) compared with OBS. After PSM (71 patients per group), survival outcomes were comparable between the two groups, with no statistical significance in RFS (HR 0.71, 95% CI 0.45–1.12, *p* = 0.14), DMFS (HR 0.73, 95% CI 0.44–1.20, *p* = 0.22), or OS (HR 1.01, 95% CI 0.58–1.78, *p* = 0.96). In conclusion, no significant survival advantage of adj‐IFN over observation was demonstrated in the matched cohort. Although a borderline association with improved RFS was observed in the overall cohort, this finding did not translate into consistent benefit across endpoints. Further prospective evidence, including the final results of the randomized phase III trial (JCOG1309, J‐FERON), is required to clarify the clinical role of adj‐IFN in NAM.

## 1 Introduction

Acral melanoma (AM) represents a distinct subtype of melanoma with a higher prevalence in Asian populations, among which nail apparatus melanoma (NAM) is a relatively uncommon but clinically challenging entity [1]. In Caucasian populations, NAM represents approximately 0.2%–2.8% of all cutaneous melanomas (CM) [2, 3, 4, 5], whereas the proportion is substantially higher in Asian populations (10%–23%) [3, 4, 5] and may reach up to 25% among African American patients [2, 3, 4, 5, 6, 7, 8]. These differences highlight the distinct epidemiological characteristics of NAM compared with other melanoma subtypes.

Compared with non‐acral CM (NACM), AM exhibits distinct genomic characteristics, including relatively low tumor mutational burden (TMB) and frequent structural genomic alterations [9]. Driver mutations commonly observed in melanoma, including *BRAF*, *NRAS*, and *NF1*, are detected less frequently in AM than in NACM, whereas alterations in *CDKN2A* and *TP53* are more prevalent [10]. As a unique subtype within AM, NAM demonstrates even more distinctive genomic characteristics, including increased copy number variations and characteristic substitution patterns, such as recurrent C/G > T/A substitutions. Whole‐exome sequencing studies have shown that NAM lacks hotspot *BRAF* and *NRAS* mutations and harbors genomic aberrations distinct from those observed in other AM subtypes. These observations suggest that NAM may represent a biologically distinct subtype of melanoma [11], raising the possibility that its response to existing therapies differs from that of other melanoma subtypes.

Although surgical resection remains the standard treatment for localized disease, the role of adjuvant therapy in NAM has not been clearly established, and evidence supporting specific postoperative strategies remains limited. Prior to the introduction of adjuvant PD‐1 inhibitors in 2018, interferon therapy had long been used as postoperative treatment for melanoma, and in Japan, IFN‐β was widely administered in the postoperative setting. In Western countries, several randomized trials evaluating IFN‐α reported modest improvements in recurrence‐free survival (RFS), particularly in patients with ulcerated primary tumors [12, 13]. However, these trials yielded inconsistent results regarding overall survival (OS), leaving its benefit controversial [12, 13, 14, 15, 16, 17, 18, 19].

In contrast, retrospective studies from Japan have suggested potential survival benefits of IFN‐β in CM populations with a high prevalence of AM [20, 21, 22]. However, none of these studies performed analyses specific to NAM, leaving the efficacy of IFN‐β in this melanoma subtype unclear. Therefore, we conducted a multicenter retrospective study to compare adjuvant IFN‐β (adj‐IFN) with observation (OBS) in patients with resected stage IIB, IIC, or III NAM.

## 2 Methods

### 2.1 Study Design and Population

The present study represents a secondary analysis of a Japanese multicenter retrospective cohort that was initially established to evaluate postoperative OBS without adjuvant therapy versus adjuvant therapy, including immune checkpoint inhibitor (ICI) therapy and IFN therapy, in patients with NAM [23]. Eligible patients were those 20 years or older who had resectable stage IIB, IIC, or III NAM confirmed by histopathological examination, treated between April 2014 and March 2024 at 42 institutions across Japan. Patients enrolled in the randomized phase III JCOG1309 (J‐FERON) trial, which compared surgery alone with adj‐IFN‐β in stage II/III melanoma, were excluded from the present analysis [24]. In addition, patients who participated in the prospective JCOG1602 (J‐NAIL) study evaluating non‐amputative digit‐preserving surgery for NAM were also excluded [25]. Primary tumors were treated with curative resection, primarily by digit amputation, with pathologically negative margins in all included cases, with sentinel lymph node biopsy performed based on clinical indications. Tumor stage was determined based on the eighth edition of the American Joint Committee on Cancer (AJCC) TNM system [26].

### 2.2 Data Collection

We collected demographic and clinicopathologic information, including patient age, sex, Breslow thickness, ulceration status, regional lymph node involvement, lymph node dissection status, postoperative management strategy (adj‐IFN or OBS), first‐line systemic therapy at recurrence, and serum lactate dehydrogenase (LDH) levels measured before the initiation of systemic therapy. The adj‐IFN cohort comprised patients who received postoperative IFN‐β therapy according to institutional practice. IFN‐β was administered locally at a dose of 3.0 × 10^6^ international units (IU) per injection, most commonly as intradermal injections around the resected primary site, with or without additional injections to regional lymph node surgical sites in patients with nodal involvement.

Although the dose per administration was consistent across institutions, treatment schedules were heterogeneous. Some institutions adopted an induction‐type regimen (e.g., daily administration for 5–10 consecutive days, repeated at predefined intervals). In contrast, other institutions initiated treatment with monthly administration without an induction phase. In patients without recurrence or metastasis, IFN‐β therapy was generally continued for approximately 2–3 years, according to institutional protocols and physician discretion.

### 2.3 Ethical Considerations

This study received approval from the central institutional review board (IRB No. 2024‐038) and was performed in compliance with the Declaration of Helsinki. Owing to its retrospective design and the use of de‐identified data, the need for informed consent was waived.

### 2.4 Outcomes

The co‐primary outcomes were RFS and distant metastasis‐free survival (DMFS). The secondary outcome was OS. RFS was calculated from the date of surgery to the first occurrence of disease recurrence or death from any cause. DMFS was defined as the interval from surgery to the development of distant metastasis or death from any cause; local and regional recurrences were not considered events. OS was defined as the interval from surgery to death from any cause. Patients without events were censored at the date of last follow‐up.

### 2.5 Statistical Analysis

Fisher's exact test was applied to categorical variables, and the Wilcoxon rank‐sum test was used for continuous data. Survival outcomes were estimated with the Kaplan–Meier approach, with group comparisons performed using the log‐rank test. Cox proportional hazards models were used to derive hazard ratios (HRs) and 95% confidence intervals (CIs). The multivariable models included adjustments for age, sex, stage, and postoperative treatment strategy.

To address potential selection bias, patients were matched using propensity scores in a 1:1 ratio with a nearest‐neighbor algorithm, applying a caliper of 0.2 times the standard deviation of the logit of the propensity score. The scores were calculated via logistic regression incorporating age, sex, and stage as covariates. In the matched cohort, Kaplan–Meier curves for RFS, DMFS, and OS were additionally generated after stratification by stage II and stage III disease. Statistical analyses were carried out with EZR version 1.68 (Saitama Medical Center, Jichi Medical University), an R‐based graphical user interface [27]. Statistical significance was defined as a two‐sided *p* value < 0.05.

## 3 Results

### 3.1 Patient Characteristics

**FIGURE 1:**
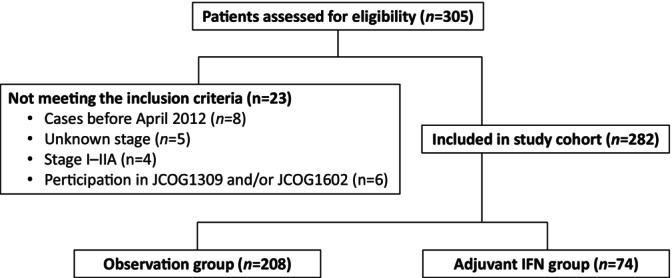
STROBE‐style cohort flowchart. Diagram of patient enrollment and allocation in the retrospective cohort study of resected nail apparatus melanoma (NAM). Among 305 patients assessed for eligibility, twenty‐three patients were excluded for the following reasons: Treatment before April 2014 (*n* = 8), unknown stage (*n* = 5), stage IA–IIA disease (*n* = 4), and participation in the JCOG trials (JCOG1309 or JCOG1602) (*n* = 6). In total, 282 patients were analyzed in either the observation (OBS) group or adjuvant interferon (adj‐IFN) group.

Among 305 patients screened for eligibility, 23 were excluded for the following reasons: treatment before April 2014 (*n* = 8), unknown stage (*n* = 5), stage IA–IIA disease (*n* = 4), and participation in the JCOG1309 or JCOG1602 trials (*n* = 6). The final study cohort therefore comprised 282 patients with resected stage IIB, IIC, or III NAM (OBS: *n* = 208; adj‐IFN: *n* = 74) (Figure 1, Table 1).

**TABLE 1:** Patient baseline characteristics.

Patient group	Total	OBS	Adj‐IFN	*p*
	*n* = 282	*n* = 208	*n* = 74	
Median age [IQR]	74.0 [65.0–81.8]	76.0 [67.0–83.0]	69.5 [61.2–78.0]	0.002
Sex, *n* (%)				
Female	109 (38.7)	81 (38.9)	28 (37.8)	0.89
Male	173 (61.3)	127 (61.1)	46 (62.2)	
Breslow thickness, *n* (%)				
< 0.8 mm	3 (1.1)	1 (0.5)	2 (2.7)	0.158
0.8–1.0 mm	1 (0.4)	1 (0.5)	0 (0.0)	
1.01–2.0 mm	8 (2.8)	5 (2.5)	3 (4.1)	
2.01–4.0 mm	89 (31.6)	71 (35.1)	18 (24.3)	
> 4.0 mm	175 (62.1)	124 (61.4)	51 (68.9)	
Ulceration, *n* (%)				
Absent	51 (18.1)	35 (16.8)	16 (21.6)	0.381
Present	231 (81.9)	173 (83.2)	58 (78.4)	
Regional lymph node involvement, *n* (%)				
Negative	176 (62.4)	138 (66.3)	38 (51.4)	0.025
Positive	105 (37.2)	69 (33.2)	36 (48.6)	
Missing	1 (0.4)	1 (0.5)	0 (0.0)	
LND for nodal involvement, *n* (%)				
Not applicable^a^	182 (64.5)	142 (68.3)	40 (54.1)	< 0.001
No	26 (9.2)	24 (11.5)	2 (2.7)	
Yes	74 (26.2)	42 (20.2)	32 (43.2)	
Stage, *n* (%)^b^				
IIB	77 (27.3)	65 (31.2)	12 (16.2)	0.023
IIC	97 (34.4)	72 (34.6)	25 (33.8)	
IIIA	4 (1.4)	2 (1.0)	2 (2.7)	
IIIB	12 (4.3)	8 (3.8)	4 (5.4)	
IIIC	77 (27.3)	54 (26.0)	23 (31.1)	
IIID	15 (5.3)	7 (3.4)	8 (10.8)	

*Note:* Data on regional lymph node involvement were missing for one patient.

Abbreviations: adj‐IFN, adjuvant interferon‐β; IQR, interquartile range; LND, lymph node dissection; *n*, number; OBS, patients without adjuvant therapy.

^a^ Not applicable includes patients with stage II disease and those with stage III disease without nodal involvement.

^b^ Tumor stage was classified according to the American Joint Committee on Cancer (AJCC) 8th edition staging system.

The median follow‐up duration was 3.6 years in the overall cohort (3.0 years in the OBS group and 5.0 years in the adj‐IFN group). The median age was 74.0 years (interquartile range [IQR], 65.0–81.8), and patients in the adj‐IFN group were significantly younger than those in the OBS group (median, 76.0 [IQR, 67.0–83.0] vs. 69.5 [61.2–78.0] years; *p* = 0.002). Among all patients, 109 (38.7%) were women and 173 (61.3%) were men, and the sex distribution did not differ significantly between the groups (*p* = 0.89).

With respect to tumor characteristics, Breslow thickness exceeded 4.0 mm in many patients (62.1%), and ulceration was present in 231 patients (81.9%), with no significant differences between the OBS and adj‐IFN groups (*p* = 0.158 and *p* = 0.381, respectively). Regional lymph node involvement was observed in 105 patients (37.6%) and was less frequent in the OBS group than in the adj‐IFN group (33.3% vs. 48.6%; *p* = 0.025). Nodal management differed significantly between the OBS and adj‐IFN groups (*p* < 0.001). A total of 182 patients (64.5%) had no nodal involvement and therefore had no indication for lymph node dissection, including 142 patients (68.3%) in the OBS group and 40 (54.1%) in the adj‐IFN group. Among patients with nodal involvement, lymph node dissection was performed in 42 (20.2%) OBS patients and 32 (43.2%) adj‐IFN patients, whereas nodal surgery was not performed in 24 (11.5%) and 2 (2.7%) patients, respectively.

Regarding disease stage, 77 patients (27.3%) had stage IIB disease, 97 (34.4%) stage IIC, 4 (1.4%) stage IIIA, 12 (4.3%) stage IIIB, 77 (27.3%) stage IIIC, and 15 (5.3%) stage IIID. The distribution of disease stage differed significantly between the OBS and adj‐IFN groups (*p* = 0.023) (Table 1).

### 3.2 Survival Outcomes in the Unmatched Cohort

**FIGURE 2:**
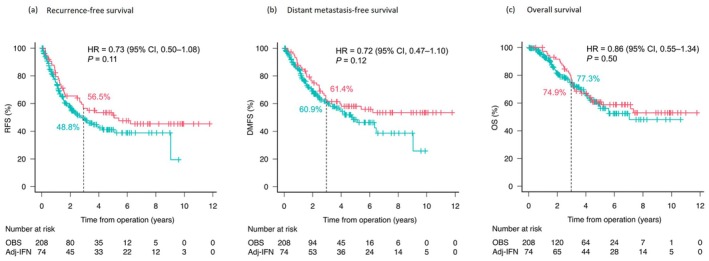
Kaplan–Meier curves for recurrence‐free, distant metastasis‐free, and overall survival in the entire unmatched cohort. Kaplan–Meier analyses of the unmatched cohort comparing the observation (OBS) and the adjuvant interferon (adj‐IFN) groups. (a) No significant difference was observed in recurrence‐free survival (RFS: HR 0.73, *p* = 0.11). (b) Distant metastasis‐free survival (DMFS) was similar across groups (HR 0.72, *p* = 0.12). (c) Overall survival (OS) also did not differ significantly (HR 0.86, *p* = 0.50).

In the entire unmatched cohort, adj‐IFN was not associated with significant improvements in RFS, DMFS, or OS compared with OBS (RFS: HR 0.73 [95% CI 0.50–1.08], *p* = 0.11; DMFS: HR 0.72 [95% CI 0.47–1.10], *p* = 0.12; OS: HR 0.86 [95% CI 0.55–1.34], *p* = 0.50) (Figure 2a–c).

### 3.3 Cox Multivariable Proportional Hazards Analyses

In multivariable Cox proportional hazards models adjusted for age, sex, disease stage, and postoperative management strategy, older age was independently associated with worse OS (HR 1.03 per year [95% CI 1.01–1.05], *p* = 0.002). Increasing disease stage was also strongly associated with poorer outcomes. Compared with stage IIB disease, stage IIC was significantly associated with worse RFS (HR 2.26 [95% CI 1.30–3.92], *p* = 0.004) and DMFS (HR 2.12 [95% CI 1.13–3.95], *p* = 0.02). Stage IIIC and IIID disease were significantly associated with worse RFS (HR 4.18 [95% CI 2.44–7.15] and HR 4.12 [95% CI 1.85–9.14], respectively, both *p* < 0.001), DMFS (HR 4.12 [95% CI 2.26–7.54], *p* < 0.001 and HR 3.79 [95% CI 1.57–9.15], *p* = 0.003), and OS (HR 2.83 [95% CI 1.51–5.32], *p* = 0.001 and HR 3.91 [95% CI 1.64–9.30], *p* = 0.002) (Table 2).

**TABLE 2:** Cox multivariable analysis in the entire unmatched cohort.

Patient group	Total	RFS		DMFS		OS	
	*n* = 282	HR (95% CI)	*p*	HR (95% CI)	*p*	HR (95% CI)	*p*
Age, per year		1.01 (0.99–1.02)	0.26	1.01 (0.99–1.02)	0.31	1.03 (1.01–1.05)	0.002
Sex, *n* (%)							
Female	109 (38.7)	Ref	—	Ref	—	Ref	—
Male	173 (61.3)	1.10 (0.76–1.58)	0.62	1.05 (0.70–1.56)	0.81	0.93 (0.60–1.44)	0.76
Stage, *n* (%)^a^							
IIB	77 (27.3)	Ref	—	Ref	—	Ref	—
IIC	97 (34.4)	2.26 (1.30–3.92)	0.004	2.12 (1.13–3.95)	0.02	1.40 (0.71–2.73)	0.33
IIIA	4 (1.4)	0.85 (0.11–6.46)	0.87	1.06 (0.14–8.27)	0.95	1.23 (0.16–9.68)	0.84
IIIB	12 (4.3)	1.84 (0.68–4.99)	0.23	2.23 (0.80–6.23)	0.13	1.23 (0.35–4.34)	0.75
IIIC	77 (27.3)	4.18 (2.44–7.15)	< 0.001	4.12 (2.26–7.54)	< 0.001	2.83 (1.51–5.32)	0.001
IIID	15 (5.3)	4.12 (1.85–9.14)	< 0.001	3.79 (1.57–9.15)	0.003	3.91 (1.64–9.30)	0.002
Treatment, *n* (%)							
OBS	208 (73.8)	Ref	—	Ref	—	Ref	—
Adj‐IFN	74 (26.2)	0.67 (0.45–0.998)	0.049	0.66 (0.42–1.01)	0.06	0.85 (0.54–1.35)	0.50

Abbreviations: adj‐IFN, adjuvant interferon‐β; CI, confidence interval; DMFS, distant metastasis‐free survival; HR, hazard ratio; *n*, number; OBS, patients without adjuvant therapy; OS, overall survival; Ref, reference; RFS, recurrence‐free survival.

^a^ American Joint Committee on Cancer 8th edition.

In contrast, adj‐IFN showed an association with improved RFS (HR 0.67 [95% CI 0.45–0.998], *p* = 0.049). No significant associations were observed for DMFS (HR 0.66 [95% CI 0.42–1.01], *p* = 0.06) or OS (HR 0.85 [95% CI 0.54–1.35], *p* = 0.50) (Table 2).

### 3.4 Survival Outcomes in the Matched Cohort

**FIGURE 3:**
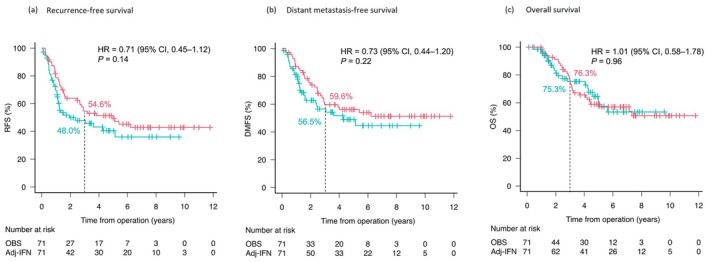
Kaplan–Meier curves for recurrence‐free, distant metastasis‐free, and overall survival in the matched cohort. Kaplan–Meier analyses of the matched cohort comparing the observation (OBS) and the adjuvant interferon (adj‐IFN) groups. (a) No significant difference was observed in recurrence‐free survival (RFS: HR 0.71, *p* = 0.14). (b) Distant metastasis‐free survival (DMFS) was similar across groups (HR 0.73, *p* = 0.22). (c) Overall survival (OS) likewise did not differ significantly (HR 1.01, *p* = 0.96).

After PSM, 71 matched pairs were identified (OBS: *n* = 71; adj‐IFN: *n* = 71). Although age and sex were well balanced between the two groups, some residual imbalance remained in stage distribution (Table 3). In the matched cohort, survival outcomes were comparable between OBS and adj‐IFN (RFS: HR 0.71 [95% CI 0.45–1.12], *p* = 0.14; DMFS: HR 0.73 [95% CI 0.44–1.20], *p* = 0.22; OS: HR 1.01 [95% CI 0.58–1.78], *p* = 0.96) (Figure 3a–c).

**TABLE 3:** Patient baseline characteristics after propensity score matching.

Patient group	Total	OBS	Adj‐IFN	SMD
	*n* = 142	*n* = 71	*n* = 71	
Median age [IQR]	70.0 [61.0–79.0]	70.0 [60.0–79.0]	71.0 [61.5–78.5]	0.02
Sex, *n* (%)				
Female	54 (38.0)	27 (38.0)	27 (38.0)	0.00
Male	88 (62.0)	44 (62.0)	44 (62.0)	
Stage, *n* (%)^a^				
IIB	26 (18.3)	14 (19.7)	12 (16.9)	0.298
IIC	46 (32.4)	21 (29.6)	25 (35.2)	
IIIA	2 (1.4)	0 (0.0)	2 (2.8)	
IIIB	8 (5.6)	5 (7.0)	3 (4.2)	
IIIC	48 (33.8)	25 (35.2)	23 (32.4)	
IIID	12 (8.5)	6 (8.5)	6 (8.5)	

*Note:* SMD for the stage reflects the overall imbalance across stage categories.

Abbreviations: adj‐IFN, adjuvant interferon‐β; IQR, interquartile range; *n*, number; OBS, patients without adjuvant therapy.

^a^ American Joint Committee on Cancer 8th edition.

Additional stage‐stratified survival analyses were performed for stage II and stage III patients in the matched cohort (Tables S1 and S2). In the matched stage II subgroup, adjuvant IFN‐β significantly improved RFS compared with observation (HR 0.39 [95% CI 0.17–0.87], *p* = 0.02), whereas no statistically significant differences were observed for DMFS or OS (Figure S1). In the matched stage III subgroup, no significant differences were observed between the two groups in RFS, DMFS, or OS (Figure S2).

## 4 Discussion

In this multicenter cohort study, adj‐IFN showed a marginal association with improved RFS in the overall cohort, whereas no significant benefits were observed for DMFS or OS in Japanese patients with resected stage IIB, IIC, or III NAM. However, this association was attenuated after PSM, and no survival advantage was observed overall. Collectively, these findings suggest that a definitive clinical benefit of adj‐IFN in NAM cannot be established. In the stage‐stratified analyses of the matched cohort, adjuvant IFN‐β was associated with improved RFS in patients with stage II disease, whereas no significant benefit was observed for DMFS or OS, and no survival benefit was observed in patients with stage III disease. Although this stage II‐specific RFS signal may be clinically relevant, it should be interpreted cautiously because of the exploratory nature of the subgroup analysis and the limited sample size.

Overall, IFN‐based adjuvant therapy has shown inconsistent and generally modest efficacy in cutaneous melanoma, raising questions regarding its applicability to NAM. In the United States, all major randomized trials of IFN‐α were conducted predominantly in patients with NACM, before the advent of ICIs. ECOG E1684 demonstrated a significant OS benefit compared with OBS (HR 0.74 [95% CI 0.55–0.99], *p* = 0.02) [19], leading to regulatory FDA approval of high‐dose IFN‐α. However, subsequent trials (E1690, E1694, and E2696) failed to reproduce this benefit, and a pooled analysis of these four ECOG studies confirmed the absence of an OS advantage (HR 0.91 [95% CI 0.77–1.07], *p* = 0.24) [18].

Similarly, the EORTC 18991 trial evaluating pegylated IFN‐α showed an improvement in RFS but no OS benefit (RFS: HR 0.82 [95% CI 0.71–0.96], *p* = 0.01; OS: HR 0.98 [95% CI 0.82–1.16], *p* = 0.78) [16]. Collectively, these findings suggest that IFN‐α‐based adjuvant therapy does not confer a sustained survival advantage.

In Japan, IFN‐β has mainly been investigated in retrospective cohort studies that included substantial proportions of AM, including NAM. Yanagi et al. reported favorable survival outcomes in a single‐institution retrospective study (RFS: HR 0.08 [95% CI 0.02–0.28], *p* < 0.001; OS: HR 0.09 [95% CI 0.02–0.39], *p* = 0.001), using a different adjuvant regimen (3 × 10^6^ IU per day on days 1–10, three to four times annually, up to 5 years). However, both the adj‐IFN and OBS groups included patients treated with DAV (dacarbazine + nimustine + vincristine)‐IFN therapy, precluding isolation of the independent effect of IFN‐β [21]. A nationwide multicenter retrospective study further evaluated several IFN‐β–based adjuvant strategies in patients with thick CM and reported improved survival only with maintenance‐type IFN‐β (OS: HR 0.34 [95% CI 0.17–0.67], *p* = 0.0022), whereas short‐course IFN‐β and DAV‐IFN did not demonstrate clinical benefit [22]. Importantly, none of these studies performed subgroup analyses specific to NAM, leaving the efficacy of IFN‐β in this melanoma subtype unresolved.

This study reflects real‐world use of adjuvant IFN‐β therapy outside a clinical trial setting rather than evaluation of the standardized JCOG1309 [24] regimen, which consists of a 6‐month induction phase followed by 2.5 years of maintenance therapy. JCOG1309 is the only prospective randomized study evaluating locoregional IFN‐β in stage II/III melanoma, and its final survival results have not yet been published. However, because the production and supply of IFN‐β have been discontinued in Japan, further clinical use of IFN‐β for melanoma is unlikely to expand under current Japanese practice. Therefore, the forthcoming results of JCOG1309 will be important for interpreting the efficacy and historical role of IFN‐β and for contextualizing adjuvant treatment strategies in melanoma.

The limited efficacy of IFN in NAM may be explained by several biological characteristics. AM is characterized by low TMB, fewer ultraviolet‐related mutations, and a high degree of structural genomic complexity, with NAM representing an extreme end of this biological spectrum [9, 10, 11]. NAM exhibits pronounced copy number alterations and characteristic substitution patterns, suggesting intrinsically low immunogenicity [10, 11]. Consistent with this biology, several retrospective studies have reported limited efficacy of anti‐PD‐1 antibodies in NAM, despite their established role as standard systemic therapy in melanoma [23, 28]. These observations may reflect a broader resistance to immune‐mediated therapeutic approaches in this melanoma subtype. This inherent low immunogenicity may also reduce the effectiveness of cytokine‐based immunomodulatory approaches such as IFN, potentially explaining the modest and non‐significant trends observed in the present study. However, the relationship between these biological characteristics and responsiveness to IFN‐based therapy remains incompletely understood.

This study has several limitations. First, despite the use of PSM, imbalance in stage persisted after matching (SMD > 0.2). As stage is a key factor of prognosis in melanoma, this residual imbalance may have confounded the survival analyses and should be considered when interpreting the matched results. In addition, although stage‐stratified analyses were performed, potential imbalances in background characteristics, particularly within stage III, may still have resulted in residual confounding. Second, although this represents the largest NAM cohort reported to date, the sample size after matching may still be insufficient to detect small but clinically meaningful survival differences. Third, central pathology review was not performed, which may have resulted in inter‐institutional variability in staging and histologic interpretation. Fourth, detailed toxicity data were not available, precluding a comprehensive assessment of IFN‐β safety. Finally, the IFN‐β treatment regimens were not standardized across institutions, with substantial variability in treatment schedules, duration, and injection sites. This heterogeneity may have diluted potential therapeutic effects and limits the interpretability of the observed survival outcomes.

In conclusion, this study did not demonstrate a significant survival advantage of adjuvant IFN‐β over observation in the propensity score–matched cohort of patients with resected NAM. Although a borderline improvement in RFS was observed in the overall cohort, this finding was not consistent across endpoints and did not translate into improved DMFS or OS. Considering the discontinuation of IFN‐β supply in Japan, routine clinical use of adjuvant IFN‐β for NAM is unlikely to expand and cannot be strongly recommended based on currently available data. The final results of JCOG1309 will nevertheless be valuable for clarifying the historical role of IFN‐β and for understanding adjuvant immunomodulatory approaches in melanoma.

## Funding

This work was supported in part by the National Cancer Center Research and Development Fund (2023‐J‐03 and 2026‐J‐03) and the Japan Agency for Medical Research and Development (AMED) (JP21ck0106681h0003).

## Ethics Statement

The requirement for informed consent was waived due to the retrospective design and use of anonymized data. The study was approved by the central institutional review board (IRB No. 2024‐038).

## Conflicts of Interest

Toshihiro Takai and Takamichi Ito are Editorial Board members of *The Journal of Dermatology* and co‐authors of this article. To minimize bias, they were excluded from all editorial decision‐making related to the acceptance of this article for publication. Dr. Hiroshi Kato receiving speaker's bureau from Ono Pharmaceutical, Novartis, and Merck Sharpe and Dohme (MSD). Dr. Tatsuya Takenouchi receiving speaker's bureau from Ono Pharmaceutical, Novartis, Bristol‐Myers Squibb (BMS), and MSD; and serving on the advisory boards of Novartis. Dr. Toshihiro Takai receiving speaker's bureau from Ono Pharmaceutical. Dr. Takeru Funakoshi receiving honoraria from Maruho; receiving speaker's bureau from Ono Pharmaceutical, Novartis, BMS, MSD, Maruho, and Kyowa Kirin International; and receiving a grant from Ono Pharmaceutical. Dr. Takayuki Suyama receiving speaker's bureau from Ono Pharmaceutical, Novartis, Maruho, and Amgen. Dr. Hiroshi Uchi receiving speaker's bureau from Ono Pharmaceutical and MSD. Dr. Jun Asai receiving speaker's bureau from Ono Pharmaceutical and MSD. Dr. Takeo Maekawa receiving speaker's bureau from Ono Pharmaceutical, Novartis, and BMS. Dr. Atsushi Otsuka has received research, speaking, and/or consulting support from BMS, Kyowa Kirin, LEO Pharma, Maruho, MSD, Novartis, Ono Pharmaceutical, Sanofi, Sun Pharma, AbbVie, and Eli Lilly. Dr. Dai Ogata receiving a grant from MSD. Dr. Kenjiro Namikawa received honoraria from Ono Pharmaceutical, Novartis, BMS, MSD, Kaken Pharmaceutical, and LEO Pharma; served as an advisory board for Novartis, MSD, Rakuten Medical, Chugai Pharmaceutical, and Taiho Pharmaceutical; received institutional research grants from Novartis, BMS, PAREXEL International, Takara Bio, MSD, Chugai Pharmaceutical, and Toray Industries. Dr. Yasuhiro Nakamura has received research grant, speaking, and/or consulting support from Alexion Pharma, BMS, Dai‐ichi Sankyo, HUYABIO International, Kyowa Kirin, LEO Pharma, Maruho, MSD, Novartis, Ono Pharmaceutical, Pierre Fabre, Regeneron, Sanofi, Sun Pharma, Tanabe‐Mitsubishi Pharma, and Toray Industries. Dr. Shigeto Matsushita receiving speaker's bureau from Ono pharmaceutical, Novartis, BMS and MSD. No other disclosures were reported.

## Supporting information


**Figure S1:** Recurrence‐free, distant metastasis‐free, and overall survival in the stage II subgroup of the matched cohort. Kaplan–Meier analyses of the matched stage II subgroup comparing the observation (OBS) and the adjuvant interferon (adj‐IFN) groups. (a) A significant difference was observed in recurrence‐free survival (RFS: HR 0.39, *p* = 0.02). (b) No significant difference was found for distant metastasis‐free survival (DMFS: HR 0.45, *p* = 0.09). (c) Overall survival (OS) likewise did not differ significantly (HR 0.80, *p* = 0.66).
**Figure S2:** Recurrence‐free, distant metastasis‐free, and overall survival in the stage III subgroup of the matched cohort. Kaplan–Meier analyses of the stage III subgroup of the matched cohort comparing the observation (OBS) and the adjuvant interferon (adj‐IFN) groups. (a) No significant difference was observed in recurrence‐free survival (RFS: HR 0.89, *p* = 0.70). (b) Distant metastasis‐free survival (DMFS) did not differ significantly between groups (HR 0.66, *p* = 0.19). (c) Overall survival (OS) likewise showed no significant difference (HR 0.83, *p* = 0.59).

**Table S1:** Patient baseline characteristics after propensity score matching in patients with stage II disease.
**Table S2:** Patient baseline characteristics after propensity score matching in patients with stage III disease.

## Data Availability

The data that support the findings of this study are available from the corresponding author upon reasonable request.
