# Supplementary material for: Efficacy of Adjuvant Interferon‐β Versus Observation for Resected Nail Apparatus Melanoma: A Multicenter Retrospective Study
Source: J Dermatol. 2026 Jul 6;53(8):1124–32. doi: 10.1111/1346-8138.70375 (PMC13435012; doi:10.1111/1346-8138.70375)
Supplement: Supplementary file 1 [file JDE-53-1124-s001.docx]

Figure S1

(a)

Recurrence-free survival

(b)

Distant metastasis-free survival

(c)

Overall survival

Figure S2

(a)

Recurrence-free survival

(b)

Distant metastasis-free survival

(c)

Overall survival
