# Supplementary material for: Efficacy of Adjuvant Interferon‐β Versus Observation for Resected Nail Apparatus Melanoma: A Multicenter Retrospective Study
Source: J Dermatol. 2026 Jul 6;53(8):1124–32. doi: 10.1111/1346-8138.70375 (PMC13435012; doi:10.1111/1346-8138.70375)
Supplement: Supplementary file 2 [file JDE-53-1124-s002.docx]

Table S1: Patient baseline characteristics after propensity score matching in patients with stage II disease.

| Patient group | Total  n = 72 | OBS  n = 36 | Adj-IFN  n = 36 | SMD |  |
| --- | --- | --- | --- | --- | --- |
|  |  |  |  |  |  |
| Median age [IQR] | 68.0 [57.0–78.0] | 68.5 [57.0–78.0] | 67.0 [57.0–78.25] | 0.04 |  |
| Sex, n (%) | | | | |  |
| Female | 29 (40.3) | 15 (41.7) | 14 (38.9) | 0.06 |  |
| Male | 43 (59.7) | 21 (58.3) | 22 (61.1) |  |  |
| Stage, n (%)^†^ | | | | |  |
| IIB | 25 (34.7) | 13 (36.1) | 12 (33.3) | 0.06 |  |
| IIC | 47 (65.3) | 23 (63.9) | 24 (66.7) |  |  |

n, number; OBS, patients without adjuvant therapy; adj-IFN, adjuvant interferon-β; IQR, interquartile range; SMD, standardized mean difference. SMD for the stage reflects the overall imbalance across stage categories.

^†^American Joint Committee on Cancer 8^th^ edition

Table S2: Patient baseline characteristics after propensity score matching in patients with stage III disease.

| Patient group | Total  n = 64 | OBS  n = 32 | Adj-IFN  n = 32 | SMD |  |
| --- | --- | --- | --- | --- | --- |
|  |  |  |  |  |  |
| Median age [IQR] | 71.0 [61.0–79.0] | 70.5 [60.0–79.0] | 72.0 [65.0–78.5] | 0.15 |  |
| Sex, n (%) | | | | |  |
| Female | 23 (35.9) | 12 (37.5) | 11 (34.4) | 0.07 |  |
| Male | 41 (64.1) | 20 (62.5) | 21 (65.6) |  |  |
| Stage, n (%)^†^ | | | | |  |
| IIIA | 2 (3.1) | 1 (3.1) | 1 (3.1) | 0.25 |  |
| IIIB | 8 (12.5) | 5 (15.6) | 3 (9.4) |  |  |
| IIIC | 46 (71.9) | 23 (71.9) | 23 (71.9) |  |  |
| IIID | 8 (12.5) | 3 (9.4) | 5 (15.6) |  |  |

n, number; OBS, patients without adjuvant therapy; adj-IFN, adjuvant interferon-β; IQR, interquartile range; SMD, standardized mean difference. SMD for the stage reflects the overall imbalance across stage categories.

^†^American Joint Committee on Cancer 8^th^ edition
